# Age-related shifts in mental health determinants from a deprived area in the European Union: informing the national healthy aging program of Hungary

**DOI:** 10.1007/s11357-024-01182-4

**Published:** 2024-05-07

**Authors:** Nora Kovacs, Eva Biro, Peter Piko, Zoltan Ungvari, Roza Adany

**Affiliations:** 1 Department of Public Health and Epidemiology, Faculty of Medicine, University of Debrecen, Debrecen, Hungary; 2 HUN-REN–UD Public Health Research Group, Department of Public Health and Epidemiology, Faculty of Medicine, University of Debrecen, Debrecen, Hungary; 3 National Laboratory for Health Security, Center for Epidemiology and Surveillance, Semmelweis University, Budapest, Hungary; 4 International Training Program in Geroscience, Doctoral School of Basic and Translational Medicine/Department of Public Health, Semmelweis University, Budapest, Hungary; 5 Vascular Cognitive Impairment and Neurodegeneration Program, Oklahoma Center for Geroscience and Healthy Brain Aging, Department of Biochemistry and Molecular Biology, University of Oklahoma Health Sciences Center, Oklahoma City, OK USA; 6 Department of Health Promotion Sciences, College of Public Health, University of Oklahoma Health Sciences Center, Oklahoma City, OK USA; 7 Department of Public Health, Semmelweis University, Budapest, Hungary

**Keywords:** Life satisfaction, Psychological distress, Mental well-being, Elderly, Ageing, Social support

## Abstract

Mental disorders are among the leading causes of disability worldwide, disproportionately affecting older people. This study aims to assess the mental health of elderly individuals living in a deprived region of Hungary, and to identify and estimate the weight of different determinants of mental health across different age groups. A cross-sectional study was conducted with randomly selected samples of individuals (n = 860) aged 18 years and older in Northeast Hungary. The World Health Organization Well-Being Index (WHO-5), the single-item Life Satisfaction Scale, and the 12-item General Health Questionnaire (GHQ-12) were used to measure mental health of the participants. Multiple linear regression analysis was performed to measure the association between sociodemographic and health-related variables and mental health. Overall, the mean WHO-5 score was 69.2 ± 18.1 and it showed a significant decrease by age (*p* < 0.001), with the lowest score observed in aged 75 years and above (*p* < 0.001). The mean life satisfaction score was 7.5 ± 1.9 and it showed a significant decreasing trend over the life course (*p* < 0.001). The highest level of psychological distress as assessed by GHQ-12 was observed in the group aged 75 years or older (11.5 ± 6.0, *p* < 0.001). Multiple linear regression indicated that self-reported financial status, social support, sense of control over their health, activity limitation and pain intensity were the most important determinants of mental health among older adults. Interventions to improve the mental health of older adults should focus on the positive impact of social support, the reduction of financial insecurity and the use of effective pain relief medications.

## Introduction

The proportion of elderly population in society has increased rapidly around the world in recent decades mainly as a result of increasing life expectancy, improved health and declining fertility rates. Globally, the number of people aged 65 years and older is expected to more than double, from 761 million in 2021 to 1.6 billion in 2050 [1]. Aging is associated with an increasing prevalence of disability, and many physical and psychological disorders [2] often challenging for the elderly.

Mental disorders are one of the leading causes of disability worldwide [3]. Since the number of people suffering from mental illnesses is expected to increase with population ageing, the mental health of older people is becoming a growing public health concern. Approximately 14% of adults aged 60 and above lived with mental disorders in 2019, accounting for 10.6% of all disability-adjusted life years (DALYs) for people in this age group [3, 4]. While mental health issues can occur at any time during the life course, older individuals are more vulnerable to mental disorders due to several factors such as social isolation [5, 6] and loneliness [6, 7], chronic diseases [8], functional disability [9] and socioeconomic factors [10]. Psychological well-being is closely related to older people’s ability to perform basic activities of daily living and maintain quality of life. Although morbidity and mortality from certain physical conditions are higher in older people with mental illness [11, 12], mental health problems are often underdiagnosed and undertreated among them [4, 13].

Family and household patterns are also changing throughout the European Union (EU), with more elderly individuals living alone [14]. Studies have demonstrated that social support and increased social participation among the elderly have protective effect against anxiety and depressive symptoms [15–17]. Moreover, social support and diversity of social networks have a significant impact on older adults’ satisfaction with their lives [18, 19].

In Hungary, the population of people aged 65 years and above has increased by 4% in the last 10 years (from 17 to 21%), reaching 2 million people in 2022 [20]. This significant growth requires the country to meet the economic, social, health, as well as psychological needs of its elderly population. Mental health problems affect approximately 5.5% of the Hungarian population [20], and it is increasing with age. In 2019 the proportion of those reported depressive symptoms among those aged 75 years and older were almost twice as high (12.4%) as in the 65–74 years age group (6.3%) [21]. In terms of life satisfaction, Hungary is one of worst performers among EU member states: in 2022, the average life satisfaction score was the fourth and fifth lowest for people aged 65–74 and 75 years and over, respectively [14]. Moreover, in 2018, the country reported the second lowest proportion of people who were highly satisfied with their lives (just behind Bulgaria) in the EU-27, both for those aged 65–74 years (8.6%) and for those aged 75 and over (6%) [21]. Despite these alarming trends, comprehensive studies on the quality of life, including mental well-being of Hungarian elderly, especially those living in deprived areas, remain scarce. A previous cross-sectional study reported a particularly poor mental health among residents of segregated settlements in Hungary, but this research was limited to individuals aged 18–64 years [22]. However, some studies have found strong association between area-level social deprivation and mental well-being among older people [23–25], which may be explained by the limited availability of financial and social resources to support residents in disadvantaged areas [25] and the neighbourhood conditions that make it difficult for individuals to maintain supportive social networks [25, 26]. Therefore, in addition to assess the psychological distress experienced by older adults living in disadvantaged areas, it is crucial to understand factors contributing to their poor mental health when developing interventions. The aim of this study was to examine the mental health of elderly individuals living in one of the deprived regions of Hungary, as well as to identify potential factors which may influence mental health across the life course.

## Methods

### Study design and sample

Data from two cross-sectional surveys carried out in 2018 and 2022 were used for the present study. Study participants for each survey were recruited from two counties in Northeast Hungary (Hajdú-Bihar and Szabolcs-Szatmár-Bereg), which is documented as one of the deprived regions of the country. Data in the first (2018) survey were collected from individuals aged 18–64 years. Twenty-five randomly selected individuals from the patients’ lists of 20 general practitioners who were involved in the General Practitioners Morbidity Sentinel Stations Program (GPMSSP), a population-based disease registry [27], were invited to participate in the study. In the second survey (2022), data were collected from individuals aged 65 years and older registered with 19 randomly selected general practices in the same counties. From each practice, 25 individuals were randomly selected and invited to participate in the survey.

Both surveys were based on a three-pillar comprehensive health assessment consisting of three main components: questionnaire-based interviews, physical examinations, and laboratory examinations. Detailed descriptions of the study design and data collection for both surveys have been previously published [28, 29]. The study sample consisted of 860 individuals of whom 417 individuals were recruited for the first survey and 443 for the second survey. The studies were conducted in accordance with the guidelines of the Declaration of Helsinki and approved by the Ethics Committee of the Hungarian Scientific Council on Health (Reference No.: 61327–2017/EKU and IV/3351–3/2022/EKU). Written informed consent was obtained from all participants in each study population before the start of the studies.

### Sociodemographic and health-related variables

The Hungarian version of the European Health Interview Survey [30] was used to collect data on sociodemographic and health-related characteristics of the participants in both surveys. Sociodemographic characteristics included age, sex, education level (primary or less, secondary, and tertiary), self-reported financial status (very good/good, satisfactory, or bad/very bad), and marital status (married, widow, other including single and divorced).

Health-related variables included reported limitations in daily activities in the past six months (limited, not limited), self-reported number of chronic diseases (less than two, two or more), and smoking status (current, former and never smoked). Participants were asked how much they could do for their own health (less/nothing and a lot/very much) to assess their sense of control over their health, and they were also asked to rate the level of pain they had experienced in the past four weeks (none, very mild/mild/moderate, or severe/extreme severe).

The social support of the participants was measured using the Oslo Social Support Scale (OSSS-3) and an assessment of support in personal matters. The OSSS-3 is a 3-item self-report measure of social support [31, 32]. The total score, ranging from 3 to 14, was calculated by summing the raw scores for the three questions, with higher scores reflecting greater social support. The level of social support was classified into three categories based on the total score: poor (3–8), moderate (9–11) and strong (12–14) social support [33, 34]. The internal consistency (Cronbach’s alpha) for this scale in the present study was 0.55. Support in personal matters was assessed by asking the respondent if they could discuss personal matters with someone (yes or no).

### Measures of mental health

The current study focuses on three outcome measures of mental health: well-being, life satisfaction, and psychological distress.

### Well-being index (WHO-5)

Well-being of participants was measured using a self-report measure (5-item World Health Organization Well-Being Index, WHO-5) developed by the World Health Organization [35]. A 6-point Likert scale ranging from 0 (not at all present) to 5 (almost always present) was used to assess the mental well-being over the previous two weeks. The raw scores were summed and multiplied by 4 to obtain a score ranging from 0 to 100, with higher scores reflecting higher levels of well-being. A score < 50 indicates poor emotional well-being and suggests further investigation [35]. Cronbach’s alpha revealed a high internal consistency for this scale (0.86) in the present study.

### Single-item Life Satisfaction Scale (LS)

Life satisfaction was assessed using a single-item, which shows comparable results to multiple-item measures [36]. Participants were asked to rate their overall life satisfaction on a scale ranging from 0 (not at all satisfied to 10 (completely satisfied) with higher scores indicating greater life satisfaction.

### General Health Questionnaire (GHQ)

The 12-item version of the General Health Questionnaire (GHQ-12), a widely used screening tool for psychological distress in the general population [37] was used to assess general mental health of participants. Using the Likert scoring system, a total score was calculated as the sum of each item scored on a four-point Likert scale (ranging from 0 to 3), with higher scores representing greater levels of psychological distress. In the present study, the Cronbach’s alpha for this scale was 0.90.

### Statistical analysis

The characteristics of the study sample were described using proportions for categorical variables and means with standard deviation and minimum and maximum values for continuous variables by age group. Because we were interested in the pattern of mental health status and social support among the participants of different ages we analysed each variable by age group. Means were calculated for each scale of mental health measures and were compared by four age groups to assess change over the life course (18–44, 45–64, 65–74, 75 and over). Descriptive statistics were performed using Pearson’s chi-squared test for categorical variables and Mann–Whitney U test and Kruskal–Wallis test for non-normally distributed continuous variables.

Multiple linear regression analysis was performed to measure the association between sociodemographic and health-related variables and continuous outcome measures of mental health status. Bootstrapping was employed in regression analyses with 1,000 resamples (due to non-normal distributions). To allow comparisons between younger and older populations, a stratified analysis was performed for age-groups of 18–64 and 65 and over. The results were presented as coefficients (b) with their corresponding 95% confidence intervals (CI). Statistical significance was set at a p-value of less than 0.05. All analyses were performed with Stata version 13.0 software (Stata Corp., College Station, TX, USA).

## Results

### Sociodemographic characteristics and (mental) health of the study population

Table 1 shows the sociodemographic characteristics of the study population by age group. The mean age of the participants was 58.8 ± 17.4 years (n = 860). The proportion of females in the 65 + age group was slightly higher than in the 18–64 age group (62.3% vs. 55.6%, *p* = 0.047). Older people had a significantly lower level of education (*p* < 0.001), reported a lower financial status (*p* = 0.038), and perceived a lower level of control over their health (*p* = 0.012). The proportion of former and never smokers (*p* < 0.001) and widows were significantly higher (*p* < 0.001) among aged 65 years and older. Health-related variables differed significantly between age groups; a significantly higher number of chronic diseases (*p* < 0.001), a higher level of pain (*p* < 0.001) and limitations in activities of daily living (*p* < 0.001) were reported by those aged 65 years and older. (Table 1).

**Table 1:** Sociodemographic characteristics and health status of the study population by age group

Characteristics		Age groups		*p*-value
	Total (n = 860)	18–64 years (n = 417)	65 + years (n = 443)	
	n (%)	n (%)	n (%)	
Sex				
Male	352 (40.9%)	185 (44.4%)	167 (37.7%)	**0.047**
Female	508 (59.1%)	232 (55.6%)	276 (62.3%)	
Age (mean ± SD)	58.8 ± 17.4	43.9 ± 12.6	72.8 ± 5.9	** < 0.001**^**a**^
Age groups				
18–44	208 (24.2%)	208 (49.9%)		
45–64	209 (24.3%)	209 (50.1%)		
65–74	291 (33.8%)		291 (65.7%)	
75 +	152 (17.7%)		152 (34.3%)	
Education level				
Primary or less	258 (30.1%)	89 (21.4%)	169 (38.3%)	** < 0.001**
Secondary	464 (54.2%)	251 (60.5%)	213 (48.3%)	
Tertiary	134 (15.7%)	75 (18.1%)	59 (13.4%)	
Self-reported financial status				
Good/very good	234 (27.8%)	130 (31.8%)	104 (24.0%)	**0.038**
Satisfactory	505 (60.1%)	230 (56.4%)	275 (63.5%)	
Bad/very bad	102 (12.1%)	48 (11.8%)	54 (12.5%)	
Marital status				
Married	499 (58.4%)	254 (61.2%)	245 (55.8%)	** < 0.001**
Widow	175 (20.5%)	20 (4.8%)	155 (35.3%)	
Other	180 (21.1%)	141 (34%)	39 (8.9%)	
Number of chronic diseases				
2 or more	534 (62.1%)	151 (36.2%)	383 (86.5%)	** < 0.001**
< 2	326 (37.9%)	266 (63.8%)	60 (13.5%)	
Sense of control over health				
Less/nothing	143 (16.9%)	56 (13.6%)	87 (20.0%)	**0.012**
Lot/very lot	704 (83.1%)	357 (86.4%)	347 (80.0%)	
Limitation in daily activities				
No	490 (57.7%)	305 (74.2%)	185 (42.2%)	** < 0.001**
Yes	359 (42.3%)	106 (25.8%)	253 (57.8%)	
Pain				
No	407 (47.8%)	265 (63.6%)	142 (32.7%)	** < 0.001**
Very mild/mild/moderate	349 (41.0%)	121 (29.0%)	228 (52.5%)	
Severe/extreme severe	95 (11.2%)	31 (7.4%)	64 (14.8%)	
Smoking				
Never smoked	516 (60.2%)	242 (58.3%)	274 (62.0%)	** < 0.001**
Yes	218 (25.4%)	134 (32.3%)	84 (19.0%)	
Former	123 (14.4%)	39 (9.4%)	84 (19.0%)	

^a^*p*-value from Mann–Whitney U test; all others are from chi-square. SD, standard deviation. Statistically significant results (*p* < 0.05) are shown in bold

The mean WHO-5 total score was 69.2 ± 18.1 and it showed a decrease between the 18–44 and 45–64 years age groups, followed by an increase in the 65–74 years age group, and with the lowest score observed in those aged 75 years and older (63 ± 18.5, *p* < 0.001). The prevalence of poor well-being (WHO-5 score < 50) was 14.5% in the total population with the highest prevalence among those aged 75 years and older (21.6%). The mean life satisfaction score was 7.5 ± 1.9 and it showed a significant decreasing trend over the life course (*p* < 0.001). Regarding psychological distress, the mean GHQ-12 total score was 9.7 ± 5.1 and it increased significantly with age, with the highest level of distress observed in those aged 75 years and older (11.5 ± 6.0, *p* < 0.001). The majority of participants (54.8%) reported moderate levels of social support as assessed by the OSSS-3 scale, while the proportion of those reporting high levels of social support was the highest among those aged 18–44 years (31.5%). Levels of social personal supports did not change significantly over the life course. (Table 2).

**Table 2:** Mental health and social support by age group

Mental health	Total		Age groups								
			18–44		45–64		65–74		75 years and over		
	mean ± SD	min–max	mean ± SD	min–max	mean ± SD	min–max	mean ± SD	min–max	mean ± SD	min–max	*p*-value
Well-being index (WHO-5)	69.2 ± 18.1	0–100	74.3 ± 15.3	28–100	66.5 ± 18.3	4–100	70.8 ± 18.3	0–100	63 ± 18.5	0–100	** < 0.001**
Single-item Life Satisfaction Scale (LS)	7.5 ± 1.9	0–10	8.0 ± 1.6	2–10	7.4 ± 1.9	1–10	7.4 ± 2.0	0–10	7.0 ± 2.0	2–10	** < 0.001**
Psychological distress (GHQ-12)	9.7 ± 5.1	0–36	8.6 ± 4.3	1–36	9.6 ± 4.9	0–36	9.7 ± 4.9	0–36	11.5 ± 6.0	1–34	** < 0.001**
**Social support**	n	%	n	%	n	%	n	%	n	%	*p*-value
Oslo Social Support Scale (OSSS-3)											
poor	140	16.9%	27	13.3%	44	22.2%	49	17.1%	20	14.0%	
moderate	455	54.8%	112	55.2%	107	54.0%	151	52.8%	85	59.4%	0.169
high	235	28.3%	64	31.5%	47	23.7%	86	30.1%	38	26.6%	
Support in personal matters											
yes	804	95.3%	196	96.1%	190	92.2%	280	96.9%	138	95.2%	0.103
no	40	4.7%	8	3.9%	16	7.8%	9	3.1%	7	4.8%	

Statistically significant results (*p* < 0.05) are shown in bold. WHO-5, 5-item World Health Organization Well-Being Index; GHQ-12, 12-item General Health Questionnaire; SD, standard deviation

### Factors associated with mental health

In multiple regression analysis, well-being of participants was positively associated with higher perceived control over their health (b = 7.23, *p* = 0.001), and inversely associated with higher age (b = -0.34, *p* = 0.043) among those aged 65 years and above. Lower financial status, having activity limitations and pain intensity were associated with lower well-being scores in both age groups. Perceived high level of social support increased well-being among participants regardless of age, however, the association was stronger among the older participants (aged 18–64 years: b = 5.54, *p* = 0.031; 65 years and above: b = 8.50, *p* = 0.002). A higher number of chronic diseases significantly reduced the well-being for adults aged 18–64 (b = -4.57, *p* = 0.023), but not for those aged 65 and over. (Table 3).

**Table 3:** Linear regression model for factors associated with well-being by age group

	Well-being index (WHO-5)					
	18–64 years			65 years or over		
	b	95% CI	p-value	b	95% CI	p-value
Age	-0.07	(-0.21, 0.08)	0.358	**-0.34**	**(-0.67, -0.01)**	**0.043**
Sex (ref: male)						
female	-0.04	(-3.16, 3.09)	0.982	-1.25	(-4.78, 2.29)	0.489
Education (ref: primary or less)						
secondary	1.68	(-2.55, 5.91)	0.436	-0.84	(-4.51, 2.83)	0.654
tertiary	0.93	(-3.73, 5.59)	0.697	-1.29	(-6.28, 3.70)	0.612
Self-reported financial status (ref: good/very good)						
satisfactory	**-3.56**	**(-6.75, -0.38)**	**0.028**	**-6.29**	**(-10.12, -2.46)**	**0.001**
bad/very bad	**-12.37**	**(-18.48, -6.27)**	** < 0.001**	**-12.05**	**(-17.99, -6.11)**	** < 0.001**
Marital status (ref: married)						
widow	-4.30	(-15.44, 6.83)	0.449	-1.98	(-5.75, 1.78)	0.302
other	0.98	(-2.00, 3.96)	0.519	1.81	(-3.90, 7.53)	0.534
Social support (OSSS-3) (ref: poor)						
moderate	3.85	(-0.10, 7.79)	0.056	4.23	(-0.88, 9.34)	0.104
strong	**5.54**	**(0.50, 10.58)**	**0.031**	**8.50**	**(3.11, 13.89)**	**0.002**
Support in personal matter (ref: yes)						
no	-0.14	(-5.87, 5.59)	0.962	-7.05	(-15.27, 1.17)	0.093
Number of chronic diseases (ref: < 2)						
2 or more	**-4.57**	**(-8.52, -0.63)**	**0.023**	-0.06	(-4.7, 4.58)	0.980
Sense of control over health (ref: less/nothing)						
lot/very lot	4.40	(-0.69, 9.48)	0.090	**7.23**	**(2.87, 11.58)**	**0.001**
Limitations in daily activities (ref: no)						
yes	**-6.03**	**(-10.10, -1.97)**	**0.004**	**-6.04**	**(-9.74, -2.35)**	**0.001**
Pain (ref.no)						
very mild/mild/moderate	**-7.41**	**(-10.65, -4.17)**	** < 0.001**	**-4.61**	**(-8.21, -1.01)**	**0.012**
severe/extreme severe	**-8.67**	**(-15.10, -2.25)**	**0.008**	**-13.01**	**(-19.39, -6.64)**	** < 0.001**
Smoking (ref: never smoked)						
yes	-2.65	(-6.14, 0.84)	0.136	0.57	(-3.65, 4.79)	0.791
former	0.56	(-4.07, 5.19)	0.812	0.87	(-3.00, 4.75)	0.659

Statistically significant results (*p* < 0.05) are shown in bold. WHO-5, 5-item World Health Organization Well-Being Index; OSSS-3, Oslo Social Support Scale; b, linear regression coefficient; CI, confidence interval

A significantly higher psychological distress score was associated with lack of support in personal matters (b = 3.64, *p* = 0.008), limitations in daily activities (b = 1.65, *p* = 0.001), perception of severe or extreme pain (b = 2.21, *p* = 0.039) and lower self-reported financial status (satisfactory: b = 1.18, *p* = 0.021; bad/very bad: b = 3.66, *p* = 0.004) among those aged 65 years and above. Psychological distress was significantly reduced by high levels of social support (b = -1.79, *p* = 0.022), higher perceived control over their health (b = -2.38, *p* = 0.005), and in former smokers (b = -1.40, *p* = 0.015). Among younger adults (18–64 years), higher number of chronic diseases (b = 1.74, *p* = 0.002) and mild (b = 1.17, *p* = 0.020) and severe pain (b = 4.53, *p* = 0.003) were significant determinants of higher level of psychological distress. (Table 4).

**Table 4:** Linear regression model for factors associated with psychological distress by age group

	Psychological distress (GHQ-12)					
	18–64 years			65 years or over		
	b	95% CI	*p*-value	b	95% CI	*p*-value
Age	-0.03	(-0.07, 0.02)	0.204	0.08	(-0.02, 0.19)	0.105
Sex (ref: male)						
female	0.13	(-0.71, 0.96)	0.770	0.22	(-0.76, 1.20)	0.657
Education (ref: primary or less)						
secondary	-0.20	(-1.37, 0.98)	0.744	0.59	(-0.48, 1.67)	0.280
tertiary	-0.10	(-1.41, 1.21)	0.880	0.36	(-1.05, 1.77)	0.613
Self-reported financial status (ref: good/very good)						
satisfactory	0.32	(-0.49, 1.14)	0.436	**1.18**	**(0.18, 2.18)**	**0.021**
bad/very bad	2.12	(0.00, 4.25)	0.050	**3.66**	**(1.18, 6.13)**	**0.004**
Marital status (ref: married)						
widow	1.61	(-0.91, 4.12)	0.211	0.07	(-1.16, 1.30)	0.908
other	0.20	(-0.81, 1.20)	0.702	-0.84	(-2.31, 0.64)	0.265
Social support (OSSS-3) (ref: poor)						
moderate	-0.43	(-1.56, 0.7)	0.454	-0.72	(-2.23, 0.80)	0.355
strong	-1.24	(-2.58, 0.1)	0.070	**-1.79**	**(-3.32, -0.26)**	**0.022**
Support in personal matter (ref: yes)						
no	1.91	(-0.51, 4.34)	0.122	**3.64**	**(0.93, 6.36)**	**0.008**
Number of chronic diseases (ref: < 2)						
2 or more	**1.74**	**(0.63, 2.85)**	**0.002**	0.60	(-0.47, 1.67)	0.272
Sense of control over health (ref: less/nothing)						
lot/very lot	-0.94	(-2.42, 0.54)	0.212	**-2.38**	**(-4.02, -0.73)**	**0.005**
Limitations in daily activities (ref: no)						
yes	0.83	(-0.47, 2.13)	0.210	**1.65**	**(0.67, 2.63)**	**0.001**
Pain (ref.no)						
very mild/mild/moderate	**1.17**	**(0.18, 2.16)**	**0.020**	-0.06	(-1.22, 1.09)	0.913
severe/extreme severe	**4.53**	**(1.57, 7.50)**	**0.003**	**2.21**	**(0.11, 4.31)**	**0.039**
Smoking (ref: never smoked)						
yes	0.10	(-0.80, 1.01)	0.821	-0.35	(-1.59, 0.88)	0.576
former	-0.48	(-1.82, 0.86)	0.480	**-1.40**	**(-2.51, -0.28)**	**0.015**

Statistically significant results (*p* < 0.05) are shown in bold. OSSS-3, Oslo Social Support Scale; GHQ-12, 12-item General Health Questionnaire; b, linear regression coefficient; CI, confidence interval

In terms of life satisfaction, older adults with strong level of social support (b = 0.84, *p* = 0.007) reported higher life satisfaction compared to those with poor social support. Limitations in daily activities (b = -0.66, *p* = 0.002) and severe or extreme pain (b = -0.71, *p* = 0.046) significantly decreased the older people’s life satisfaction. A higher number of chronic diseases was inversely associated with life satisfaction among 18–64 years olds (b = -0.65, *p* = 0.001). In addition, worse financial status and sense of control over their health were significant determinants of life satisfaction in both age groups. (Table 5).

**Table 5:** Linear regression model for factors associated with life satisfaction by age group

	Single-item Life Satisfaction Scale (LS)					
	18–64 years			65 years or over		
	b	95% CI	*p*-value	b	95% CI	*p*-value
Age	0.00	(-0.01, 0.01)	0.951	0.00	(-0.04, 0.03)	0.995
Sex (ref: male)						
female	-0.10	(-0.42, 0.22)	0.536	0.16	(-0.23, 0.55)	0.420
Education (ref: primary or less)						
secondary	0.33	(-0.13, 0.8)	0.159	0.05	(-0.38, 0.47)	0.826
tertiary	0.30	(-0.24, 0.85)	0.272	-0.14	(-0.74, 0.47)	0.655
Self-reported financial status (ref: good/very good)						
satisfactory	-0.34	(-0.68, 0.01)	0.055	**-1.11**	**(-1.51, -0.71)**	** < 0.001**
bad/very bad	**-1.24**	**(-1.87, -0.62)**	** < 0.001**	**-1.99**	**(-2.76, -1.22)**	** < 0.001**
Marital status (ref: married)						
widow	0.09	(-0.78, 0.96)	0.839	-0.30	(-0.77, 0.16)	0.196
other	0.11	(-0.23, 0.45)	0.517	0.53	(-0.09, 1.15)	0.091
Social support (OSSS-3) (ref: poor)						
moderate	0.24	(-0.22, 0.7)	0.306	0.54	(-0.05, 1.14)	0.075
strong	0.38	(-0.12, 0.89)	0.139	**0.84**	**(0.22, 1.45)**	**0.007**
Support in personal matter (ref: yes)						
no	-0.34	(-1.13, 0.46)	0.410	-1.10	(-2.54, 0.33)	0.132
Number of chronic diseases (ref: < 2)						
2 or more	**-0.65**	**(-1.05, -0.25)**	**0.001**	-0.06	(-0.59, 0.47)	0.830
Sense of control over health (ref: less/nothing)						
lot/very lot	**0.77**	**(0.23, 1.32)**	**0.006**	**0.59**	**(0.1, 1.08)**	**0.019**
Limitations in daily activities (ref: no)						
yes	-0.37	(-0.8, 0.07)	0.102	**-0.66**	**(-1.09, -0.24)**	**0.002**
Pain (ref.no)						
very mild/mild/moderate	-0.36	(-0.73, 0.00)	0.052	-0.22	(-0.65, 0.22)	0.324
severe/extreme severe	-0.79	(-1.64, 0.06)	0.070	**-0.71**	**(-1.41, -0.01)**	**0.046**
Smoking (ref: never smoked)						
yes	-0.24	(-0.58, 0.1)	0.175	0.27	(-0.26, 0.81)	0.313
former	-0.36	(-0.93, 0.2)	0.209	0.23	(-0.16, 0.62)	0.252

Statistically significant results (*p* < 0.05) are shown in bold. OSSS-3, Oslo Social Support Scale; b, linear regression coefficient; CI, confidence interval

## Discussion

Our study found a significant decline in mental health with increasing age among people living in Northeastern Hungary, a deprived region of the country [38]. Self-reported financial status, social support, sense of control over their health, limitations in daily activities and pain intensity were independently associated with mental health of the elderly.

Psychological well-being is one of the most important indicators of successful ageing and is strongly associated with health and longevity [39–41]. Previous studies, mainly relying on cross-sectional data, suggested a U-shaped relationship between age and well-being, with a significant decline in life satisfaction from young adulthood to midlife, followed by an increase after middle age [42–44]. However, studies based on longitudinal data have provided conflicting evidence about the changing patterns of well-being over the life course [45–47]. In our study, we found that the well-being declines between the ages of 45 and 64 years, increases over to the early seventies and declines again. Life satisfaction shows a similar pattern, however, instead of rising in middle age, it flattens out and than begins to decline among the oldest.

According to our findings, several factors contributed to the decline in mental health in later life. Previous research has demonstrated that the decline in life satisfaction in old age is mainly due to health problems and social isolation [48]. Social support is an important protective factor against mental health problems, since it can prevent loneliness and delay the development of diseases. Although the levels of social support did not change significantly over the life course in our study, high levels of social support were associated with less psychological distress, greater well-being and higher life satisfaction in the elderly, while a smaller effect on well-being was observed in younger adults. A comprehensive systematic review recently reported that the sources of social support that were most protective against depression vary across the life course and support from spouses and friends being the most important sources of social support for older adults [49]. However, others highlighted the importance of older adults' access to meaningful relationships rather than simply increasing their social interactions [50]. Lack of personal support was also associated with higher levels of psychological distress especially among those aged 65 years and over, which is in line with a previous research showing that the life stressor of “having no one to talk to” was strongly associated to the onset of depressive symptoms in later life [51]. Our findings reinforce the positive effect of social support on mental health of the elderly, as it was independent of sociodemographic variables or self-reported health problems.

Our study found that limitations in daily activities rather than the number of chronic diseases were associated with mental well-being in older age. A possible explanation can be that due to increasing life expectancy, people are much more likely to experience an increased burden and complexity of multimorbidity which ultimately lead to severe disability and functional impairment and greatly reduce their quality of life in later life [48]. As the number of adults living with disabilities increases with the aging of the population, meeting their health and support needs remains a major challenge. Restricted social participation is more common among those with impairments or functional limitations, which can affect their health and psychological well-being [52]. In addition, a recent review suggests that older adults face a range of emotional and psychological difficulties caused or exacerbated by chronic conditions that may go undetected among them [50].

Acute and chronic pain play an important role in the mental health of people of all ages. Consistent with previous findings, we found a significant relationship between pain and mental well-being in both younger and older people with adults reporting higher level of pains having poorer mental health [53, 54]. Pain management requires to address the psychiatric needs of patients with pain and comorbid mental health problems [55], since those suffering from this complex issue are more likely to report poorer health related quality of life and more disability [56, 57].

Our study revealed a significantly better psychological well-being among adults aged 65 years and above who thought they could do lot or very lot for their health. The association between perceived control and psychological well-being was not consistent in the younger population, only the life satisfaction showed significant association with greater perceived control over health. Previous studies have suggested that a greater perceived control is associated with positive health outcomes in older age [58, 59], and with better psychological well-being, fewer depressive symptoms, less loneliness, and more contact with friends [60]. Furthermore, individuals experience a decline in perceived control in later adulthood, due to the increasing prevalence of age-related barriers and constraints (e.g. health and social conditions) [61, 62]. Our finding indicates that increasing older adults’ sense of control over their health may be an independent factor in addition to social support, in achieving better physical and mental health.

Self-reported financial status was found to be a significant predictor of psychological well-being across the lifespan in the present study, and the association was even stronger among the elderly. This finding supports the current literature which has found that low levels of income are strongly associated with an increased risk of poor psychological well-being in older age [8, 63]. In addition, a previous research has shown that financial strain acts as a stressor in older people and is associated with adverse physical and mental health outcomes in later life [64].

Central and Eastern European countries are at the bottom of the Aging Society Index ranking, an evidence-based model of a successfully aging society. This suggests that these countries, and Hungary in particular, are likely to face challenges in adapting to demographic transition, particularly in the areas of well-being, productivity and engagement [65]. In order to be able to prepare better for these challenges and to plan and develop interventions, it is utmost important to have reliable information about the physical and mental health of the elderly. Several studies have reported generally poorer mental health of older adults from Central and Eastern European countries [66–68], but to the best of our knowledge, this is the first study to comprehensively assess changes in the determinants of mental health across the life course in a Central and Eastern European country.

This study is subject to several limitations. Given the cross-sectional study design, causal relationship cannot be inferred between investigated variables and psychological well-being. The use of self-reported measures of health status may be prone to recall bias, which could lead to overreporting of certain conditions. Although we have included significant socioeconomic and health-related variables in our multivariable models, additional independent variables may still have an impact on the outcomes. This study focuses on a specific disadvantaged region of Hungary, which limits the generalizability of our findings to the Hungarian population as a whole. In addition, data collection for the second survey was carried out in May–August 2022, during the COVID-19 pandemic, which may have affected participants' mental health [69, 70]. Due to COVID-19, older people may have experienced bereavement, loss of a partner or a member of their social network or reduced participation in social and public activities, which could lead to loneliness or worsening of their mental health [71]. However, we found no evidence of poorer mental health among the studied elderly compared to the findings of the European Health Interview Survey 2019, so we can assume a real, pandemic-independent difference in the mental health of the different age-groups members [72]. In addition, the data collection of the two surveys took place in different years which may moderate the effect of association and the observed trend across age-groups. However, the same methodology and use of standard questionnaires allowed a reliable comparison of the study samples.

In conclusion, our in-depth investigation of the mental health challenges faced by older adults in a socioeconomically disadvantaged region of Hungary has revealed significant disparities in well-being, life satisfaction, and psychological distress between different age groups. The complex interplay of financial status, social support, sense of control over health, limitations in daily activities, and pain intensity stands out as crucial determinants affecting the mental health of older adults. Our findings not only deepen our understanding, but are also pivotal in shaping the strategic development of the Hungarian National Healthy Aging Program, which is currently in its nascent stages.

Our collaborative team is actively engaged in the formulation of this crucial program, working closely with governmental bodies. Alongside, we are in the process of establishing a WHO Collaborating Center in partnership with the World Health Organization (WHO) Regional Office for Europe [73] drawing upon the expertise and resources of Semmelweis University. This collaboration is in step with the WHO-led United Nations Decade of Healthy Ageing (2021–2030) [74], an initiative aligned with the final decade of the Sustainable Development Goals [75] aimed at improving the lives of older people, their families, and their communities. The Decade calls upon governments, international and regional organizations, civil society, the private sector, academia, and the media to join forces and to advance the goals of this transformative era. Our efforts to develop the National Healthy Aging Program are significantly inspired by WHO's guidance and the overarching framework of the United Nations Decade of Healthy Ageing. Our research, inspired by this agenda, provides vital insights and evidence to guide the formulation of components within the National Healthy Aging Program. Our findings serve as essential baseline data, offering perspectives that can inform future health strategies and initiatives designed to enhance the well-being of the elderly in Hungary and other socioeconomically disadvantaged regions within the European Union.

By addressing the complex needs identified through our research, and by enhancing social networks and ensuring accessible, effective healthcare interventions, we can substantially improve the mental well-being of older adults. As the Hungarian National Healthy Aging Program progresses, the insights from our study will enrich the ongoing dialogue on healthy aging – a critical conversation for developing policies and programs that comprehensively meet the needs of the aging population and prioritize their mental health and overall well-being in Hungary's public health agenda. Moving forward, it is essential to continue building on these foundations, harnessing international collaboration and evidence-based strategies to create a society where healthy aging is not merely an aspiration but a reality for all.

## Data Availability

The data are available from the corresponding author upon reasonable request due to privacy or ethical concerns.
